# Characteristics of parents with a mental illness and their minor children in a study sample from the Czech Republic: a cross-sectional study

**DOI:** 10.1186/s12888-026-08118-6

**Published:** 2026-05-16

**Authors:** Adéla Farářová, Hana Papežová, Camilla Lauritzen, Charlotte Reedtz, Jana Gricová, Václav Čapek, Tereza Štěpánková, Karin van Doesum

**Affiliations:** 1 Department of Psychiatry, First Faculty of Medicine, Charles University and General University Hospital in Prague, Prague, Czech Republic; 2 Regional Center for Child and Adolescent Mental Health and Child Welfare, Faculty of Health Sciences, UiT The Arctic University of Norway, Tromsø, Norway; 3 First Faculty of Medicine, Charles University, Prague, Czech Republic; 4 Department of Clinical Psychology, Radboud University, Nijmegen, Netherlands

**Keywords:** Parental mental illness, Risk of mental disorders, Children, Family support, Prevention

## Abstract

**Background:**

The association between a parent’s mental illness and the risk of mental disorders in the offspring is multifactorial. The aim of this study was to explore the characteristics of mentally ill parents and their minor children and to provide information on family influences among families who consented to participate in a cross-sectional study. Specifically, this study explores the sociodemographic data, diagnoses of mentally ill parents and their minor children, and the perceived quality of life of the children, assessed by both parents and children themselves.

**Methods:**

Participants were 65 families with 100 children aged under 18, where one or both parents had a formal diagnosis of a mental disorder. We obtained a variety of sociodemographic and clinical data from the families/parents and their children and identified certain risk and protective characteristics. We analyzed the relationships between the observed measures (responses in questionnaires) and selected demographic and risk factors using a multivariable linear regression model. Normality of residuals was verified by the Shapiro–Wilk test. P-values < 0.05 were considered statistically significant.

**Results:**

We found that a total of 31 children from 28 families already had an ICD-10 mental disorder diagnosis. Half of the parents lived with the child’s other parent. Just over one half of the parents had been hospitalized in the past. Just under one quarter of the parents had experienced their own parents’ mental illness. Over one half of the parents had informed their offspring about their psychiatric diagnosis. The greater the number of risk factors the family faced, the worse the children perceived their quality of life. The higher the net family income was, the better both children and parents perceived the children’s physical well-being.

**Conclusions:**

Our findings highlight the need for targeted prevention for parents with mental illness and their children. Identification of adult patients who are parents is the starting point for reaching out to other family members, especially the most vulnerable, that is, the children. Targeted prevention can address multifactorial links, alleviate the impact of parental disorder on children and mitigate the risk of psychopathology in the offspring.

**Trial registration:**

ClinicalTrials.gov Identifier: NCT05554458. Registered on 16 September 2022. Retrospectively registered.

**Supplementary Information:**

The online version contains supplementary material available at 10.1186/s12888-026-08118-6.

## Background

Over the past two decades, there has been a growing emphasis on understanding the complex interplay between mental illness and parenting. This heightened focus is particularly relevant given that a significant number of adults with mental illness become parents. In adult psychiatric services, prevalence rates of parents range from 12.2% to 45.0%, with a higher proportion of mothers (34% to 59%) compared to fathers (25% to 39%). Single parent status ranges from 39% to 90% [1]. This context underscores the critical need to explore the complex dynamics between mental health issues and parenting, and to be aware of the potentially far-reaching impact on adults and their children.

Offspring of parents with a mental disorder face an increased risk of developing a mental disorder themselves, a problem highlighted by international estimates that 4–23% of children and adolescents have at least one parent with a mental disorder [2]. However, other studies have found even higher proportions of children living with parents with mental illness. The Survey of Mental Health and Wellbeing of 6,310 children and adolescents found that over one-third of 4–17-year-olds had a primary caregiver with a lifetime diagnosis. The prevalence of all disorders was significantly higher among these children than among children whose primary caregiver reported no diagnoses, and highest when the primary caregiver had comorbid and more severe disorders [3]. Another study of 152 children and adolescents attending mental health services found that 79% of these children lived with a parent with a mental illness [4].

As a result, a growing body of research is examining the co-occurrence of mental illness in both parents and children, and finding that offspring may develop the same, but also different, mental disorders [5, 6]. A comprehensive meta-analysis including 211 studies and more than 3 million offspring of parents with mental illness underscores a consistent trend of increased risk of mental disorders in children of parents with various psychiatric illnesses. The lifetime probability of developing any mental disorder is more than 50% for offspring of parents with anxiety, bipolar, and depressive disorders. The lifetime risk of offspring developing the same mental disorder that their parents were diagnosed with is highest for attention-deficit/hyperactivity disorder (32%), followed by anxiety disorders, and depression [6]. Another systematic review comparing 76 studies on child and parent diagnoses assessed that the risk transfer is partly specific. Children of anxious parents have a strong tendency to develop the same disorder as their parents. Multifinality and equifinality are characteristic for offspring of unipolar and bipolar parents [5].

Given the elevated risks, families with a parent with a mental illness (FAPMI) who are raising minor children are considered vulnerable. These families are more likely to suffer from impaired functioning [7–10]. Family functioning may be affected by a limited parenting style that is shaped by the parents’ difficult experiences as a result of mental illness. Parents’ feelings of guilt towards societal ideals of parenting, as well as their concerns about the health of their offspring, can lead to psychological distress and to strained parent–child relationships. Parents may find it difficult to reconcile their personal needs with those of their children. Although some children in families with parental mental illness assume additional responsibilities or caregiving roles, this is not universal. Children’s experiences are heterogeneous and influenced by family functioning, symptom severity, social supports, developmental stage, and context-specific factors. Many offspring show resilience and do not assume caregiving roles, while others may shoulder responsibilities that can at times exceed what is developmentally appropriate. Recognizing this variability is important to avoid overgeneralization and to ensure that prevention and support are responsive to different needs and trajectories [8, 11, 13, 24].

The risk of transmitting psychopathology from parents to the offspring is influenced by various factors, including genetics and adverse childhood experiences [5, 12]. Children of parents with a mental illness (COPMI) may become the next generation of psychiatric patients, particularly when various risk factors have a negative impact on their development [1, 2]. These factors include parent-related factors (e.g., emotional bonding with the child and parenting consistency), individual factors (such as the child’s gender, intellect, and social skills), and social factors (such as socioeconomic status and wider social networks, including the school environment) [13]. Conversely, these factors can also be protective, preventing the onset or worsening of mental health difficulties [14].

Given the awareness of increased risk, there has been an emphasis on systematic preventive work with FAPMI and COPMI, the expansion of services that respond to the needs of these groups [1, 15], and on the reduction of exposure to risk factors and promotion of protective factors [16, 17]. Studies indicate that targeted prevention that takes into account multifactorial influences can effectively reduce transgenerational transmission of psychopathology by up to 40% and even more [18, 19]. Provision of preventive interventions can not only be widely applicable but also cost-effective, regardless of the parent’s mental disorder diagnosis [13].

Mental health professionals who work with adult patients should identify the parental role of patients [20–22]. A secure, trusting, and judgement-free collaboration can enhance parents’ openness to psychoeducation about mental illness risks and increase the potential for professionals to work with their children [17, 18, 23]. This collaborative approach allows for tailored services addressing the complex needs of FAPMI and preventive interventions for COPMI [24]. Positive past experiences with mental health professionals play a crucial role in establishing effective professional contact with families [25–27].

As described above, prevention is an important area for collaboration with FAPMI and COPMI. Preventive interventions can improve parents’ awareness of the impact of mental disorders on their children and of the risks of transmitting psychopathology to the offspring. Acknowledgement of the parental challenges normalizes the parents’ feelings and experiences and aids transparent communication between children and parents about the parents’ illness [15, 25, 28, 29]. Lack of parental awareness complicates communication, affects the children’s quality of life, and contributes to their feelings of shame, guilt, and social isolation. COPMI are also vulnerable to negative peer reactions, which have an impact on their social relationships [13, 14, 30].

Parents who are unaware of the impact of their mental illness on their children’s quality of life may overlook the complexity of issues their offspring are facing. Increased awareness of these challenges and personal experience with mental disorders can enhance parental competence by enabling a better recognition of their children’s mental health problems, ultimately leading parents to seek professional care for their children [25–27]. Recognition of mental health difficulties is influenced by the children’s age and gender. Parents tend to seek help for younger boys with externalizing symptoms, while during adolescence they more often look for help for girls with internalizing problems that tend to increase with age [25, 27]. In later adolescence, recognition of mental health difficulties can be complicated because children may hesitate to burden or disappoint their parents, which leads to lack of open communication [25].

The quality of life of FAPMI and COPMI can be positively influenced by family communication, but parents often lack the communication skills needed to discuss their mental health problems [20, 21]. Preventive interventions targeting parent–child communication can alleviate parental fears of failure, judgment [15, 30], and stigma associated with mental disorders, and contribute to improved family dynamics [9, 15, 25]. Parents who associate mental illness with shame face greater challenges in breaking down the stigma [25, 28, 31, 32].

Other factors affecting children’s quality of life include parental hospitalization. While some parents recognize the benefits of psychiatric hospitalization for their mental health, they struggle with the associated loss of contact with and involvement in their child’s care. COPMI themselves have conflicting emotions: they feel grateful for the care their parents are receiving but also struggle with loneliness due to separation, worry, and fear of the hospital environment [9, 15, 30]. In families with no additional caregivers or wider family support, COPMI may face inappropriate situations and existential challenges, including financial struggles, housing insecurity, and the burden of care for younger siblings.

Additionally, COPMI whose parents have a severe mental illness that requires hospitalization are more likely to face developmental fragility in various school readiness areas irrespective of whether it is the mother or father who is hospitalized. Parental hospitalization coupled with inadequate support for early physical development contributes to motor skill difficulties and can lead to lack of independence in the classroom or in the playground [7]. COPMI are also susceptible to poor physical health and are at an increased risk of injury, asthma, malnutrition, and diarrhea compared to their peers, regardless of whether the mother or father undergoes treatment [12].

Adverse financial conditions and low socioeconomic status are critical risk factors that impact the quality of life of COPMI [31–33]. Low socioeconomic status contributes to family instability and affects various aspects of social life, including limited access to education [32]. A systematic review by Reiss [32] based on studies on socioeconomic indicators such as household income, family poverty, parental education, and occupational status found that socioeconomically disadvantaged children and adolescents were two to three times more likely to experience mental health problems. Oliver-Parra et al. [31] emphasized the impact of socially constructed gender roles in parents with mental illness, particularly for mothers with low socioeconomic status who, as primary caregivers, are associated with more mental health problems in the offspring than fathers.

For FAPMI, it is often difficult to achieve a sufficient income, especially when the parent struggles with a serious mental illness that requires repeated hospitalizations [31–33]. The partner often assumes the breadwinner role, but the associated burden can strain the relationship and potentially lead to its breakup [34]. The mental disorder itself affects the partner’s functioning and contributes to disagreements that may lead to family disintegration and further financial instability [15, 30, 34, 35].

Currently, little is known about the families with minor children in the Czech Republic in terms of their sociodemographic background, diagnoses, quality of life, and risk factors for development of mental health disorders. We have therefore carried out a cross-sectional study to document the situation of these families with the aim of creating a knowledge base for targeted preventive interventions for FAPMI/COPMI. This study extends a well-established global evidence base to a Central/Eastern European context by describing families in the Czech Republic across multiple service settings. We used a family-focused framework [13, 16, 23] to examine (a) the characteristics of families with parental mental illness and minor children; (b) the relationship between parental and child diagnoses; (c) children’s health-related quality of life from both children’s and parents’ perspectives, as well as parents’ perceived threats to healthy development in younger children; and (d) perceived financial constraints. In addition to replicating core risk-protective associations, the study provides novel, context-specific descriptive information on disclosure within families, and multi-domain financial strain that can inform tailored preventive efforts in the Czech Republic.

### Aims and research questions

Grounded in prior evidence on transdiagnostic risk transmission and family-focused prevention [5, 6, 13, 16, 18, 19, 23], our objectives were to:

- Characterize families with children under 18 in which one or both parents have a formal ICD-10 mental disorder.
- Explore the relationship between parental diagnoses and children’s diagnoses, acknowledging both specific and transdiagnostic risk pathways.
- Assess children’s health-related quality of life (HRQoL) as reported by children (≥ 12 years) and by parents (proxy reports), and examine parents’ perceived threats to healthy development in younger children.
- Describe perceived constraints in specific areas of the household budget.

These aims address a gap in evidence from Central/Eastern Europe and were guided by the Model Protocol for COPMI research [16].

## Methods/design

### Cross-sectional design

The current project was designed as a cross-sectional study. We administered questionnaires to FAPMI with the aim of collecting sociodemographic and clinical data and assessing the risk and protective characteristics. In addition to these electronic questionnaires, therapists administered specific questionnaires in person and collected them directly from the study participants. The questionnaires were completed by parents of children under 18 years of age and by children aged 12 years and above. All questionnaires were completed anonymously. The study design, procedures, and measures were approved by the Ethics Committee of the First Faculty of Medicine of Charles University and the General University Hospital in Prague, Czech Republic (under reg. no.: 1309/20 S-IV Grant). The study was conducted in the Czech Republic [36] and followed the Norwegian Arctic University model protocol, which incorporates recommended measurement tools [16].

### Participants

We approached 176 families through their therapists, who met inclusion criteria. 65 families with 100 children aged under 18, in which one or both parents had a formal diagnosis of a mental disorder, participated in the study. Eligibility criteria required that at least one parent met DSM-5 or ICD-10 criteria for a mental disorder, established by clinicians authorized to diagnose according to Czech regulations. To preserve anonymity while ensuring diagnostic validity, therapists confirmed parent and child diagnoses via a secure, de-identified linkage (study codes) after participants completed the sociodemographic questionnaire. The research team had access only to coded data; therapists retained any identifiable information at their sites. Where a child had a diagnosis, this was confirmed in the same manner.

Exclusion criteria were: acute parental substance or alcohol dependence; acute mental disorder with significant and distressing symptoms (e.g., acute psychosis, suicidality) in the parent or child requiring immediate treatment; parental inability to provide consent due to intellectual disability; and language barrier. We excluded families meeting acute criteria to minimize participant burden and ensure safety during periods of crisis; clinical care was prioritized in these cases. We did not systematically track counts per exclusion criterion across sites, which we acknowledge as a limitation.

Recruitment occurred in five planned waves between March 2021 and April 2023. Phases were scheduled to accommodate COVID-19-related service interruptions and therapist availability across participating settings: Wave 1 (Mar–Jun 2021), Wave 2 (Jul–Oct 2021), Wave 3 (Nov 2021–Feb 2022), Wave 4 (Mar–Aug 2022), and Wave 5 (Sep 2022–Apr 2023). This phased approach aimed to maximize reach across diverse institutions while minimizing participant burden during periods of heightened clinical demand.

### Procedure

Therapists were mental health professionals experienced in working with adult or child patients with mental disorders or with families where one or both parents are mentally ill. Therapists informed both parents about the study. Written informed consent was obtained from all participating adults. For children aged 12–17 who completed the child questionnaire, written informed consent was obtained from a parent or legal guardian in accordance with Ethics Committee approval. Participation was free of charge.

Of nearly 100 institutions initially contacted, 23 therapists from multiple sectors ultimately participated. Therapists represented adult psychiatric services (including state and university hospitals), child and adolescent mental health services, mental health centers, educational and psychological counselling centers, and NGOs. Families were therefore recruited from varied clinical and psychosocial contexts. We did not systematically record the exact number of families per institution; however, in our Discussion we address potential differences in burden profiles between settings as a limitation.

### Measures

The questionnaires covered the following subjects:

#### Sociodemographic and risk & protective characteristics

The questionnaire was completed by the parent with mental illness. Personal demographic variables included data on the age and gender of the parent, parent’s diagnosis (we distinguished six categories according to ICD-10 classification: F20–F29, F30–F39, F40–F48, F50–F59, F90–F98, and other diagnoses), the number of children, and the age and gender of the children. The questionnaire also investigated the socioeconomic status of the family: parent’s education (based on the years of formal education we distinguished categories of low, medium, and high), net family income, and whether the family has enough money for housing, food, clothing, toiletries, holidays, hobbies, education and leisure for the parent, and repairs.

We constructed a formative index capturing cumulative family adversity across domains recommended in preventive frameworks for COPMI [13, 16, 33]. Each domain was scored as present (1) or, for higher-severity conditions, weighted as 2; the total score ranged from 0 to 10, with higher values indicating greater cumulative risk. The index is formative by design (i.e., items define the construct rather than reflect a single latent factor), so internal consistency is not applicable. Domains and scoring were grounded in the literature on cumulative risk and in clinician consensus:

- Parental clinical severity: psychiatric comorbidity (1); frequent hospitalization (≥ 1–2 times per year) (1).
- Family psychiatric burden: at least one child with an ICD-10 mental disorder (1); at least one grandparent (parent of the respondent) with a mental disorder (1); the second caregiver with a mental disorder (1).
- Caregiving support: presence of additional caregiver(s) beyond the respondent scored as follows—no risk if a co-residing second parent shared caregiving; value 1 if caregiving support was available but did not involve a co-residing second parent (e.g., non-resident partner, grandparents, extended family); value 2 if the respondent reported no additional caregiver.
- Diagnosis awareness: second caregiver unaware of the respondent’s diagnosis (1); children unaware of the respondent’s diagnosis (1).
- Financial strain across basic and developmental needs: lack of funds reported for 1–2 domains (housing, food, clothing, toiletries, holidays, hobbies, education/leisure, repairs) (1); lack of funds in ≥ 3 domains (2).

To enhance transparency, we provide a concise overview of domains and weights in an Additional file 2 (see Supplementary Table S1). The Sociodemographic questionnaire used in this study was specifically developed for this research. An English version of the questionnaire is available as an Additional file 1.

#### KIDSCREEN-27

The 27-item KIDSCREEN measures children’s HRQoL across five subscales (Physical well-being, Psychological well-being, Autonomy and parent relations, Peers and social support, School environment) on five-point response scales; higher scores indicate better HRQoL [37, 38]. In our study, children aged 12–18 completed the self-report version, consistent with Ethics Committee guidance. For HRQoL proxy reports, parents completed the parent version for children aged 8–18. Children under 8 years were not included in KIDSCREEN analyses. Cronbach’s alpha in our sample was 0.93 for child self-reports and 0.89 for parent proxy reports.

#### Parents’ evaluation of developmental status (PEDS)

Parents of children aged 21 months to 8 years completed the PEDS (10 items) to identify developmental/behavioral concerns, yielding risk categories A (high), B (moderate), C (low), D (no concerns but risk) [39, 40]. Given the small number of returns (*n* = 18 families), we report only descriptive distributions; no inferential tests were performed for PEDS. Reliability scores have been found adequate in previous studies [16]. Cronbach’s alpha for our specific sample was 0.62.

### Data analyses

We fitted multivariable linear regression models for each KIDSCREEN-27 subscale to examine associations with pre-specified covariates: parent’s gender, age, education level, hospitalization (ever vs. never), parental diagnosis (ICD-10 category), net family income, cumulative family risk index, number of children, and presence of another adult caregiver in the household. Inclusion of both a cumulative risk index and select individual covariates was intentional. The index captures cross-domain accumulation of adversity, while certain covariates (e.g., lifetime hospitalization, co-residing caregiver, net income) reflect distinct operationalizations valuable for interpretability and adjustment. In particular, the risk index hospitalization component represented frequent hospitalizations (≥ 1–2 times/year), whereas the dichotomous covariate captured any lifetime hospitalization; caregiver support in the index differentiated a gradient of support versus a binary presence in the covariate; and multi-domain financial strain (index) was conceptually distinct from net income (covariate).

Descriptive statistics for all variables included in the regression analyses are presented in the Results section prior to the regression models to provide context for the analytical findings.

Normality of residuals was assessed by the Shapiro–Wilk test. P-values < 0.05 were considered statistically significant. Cronbach’s alpha was computed for each instrument at the scale level (i.e., all items within each instrument). Parent–child differences were calculated as child self-report minus parent proxy for dyads where both completed the corresponding subscale. Analyses were conducted in R 4.3.2 (packages: stats for linear models and Shapiro–Wilk tests; psych for reliability analyses).

Subsamples: Child self-report models included participants aged 12–18; parent proxy models included children aged 8–18. Analyses of parent–child differences were restricted to dyads with both reports available on a given subscale; we report the number of dyads per subscale in Results.

## Results

### Characteristics of families with children aged under 18 years where one or both parents have a formal diagnosis of a mental disorder

Most parents in our sample were mothers (83%), 17% were fathers. Most parents had a medium (42%) or high education (49%) level, while a smaller proportion had low education (9%). Families had on average 1.54 children (SD = 0.73). Parents with a mental illness had a mean age of 41.92 years (SD = 6.98), with mothers at a mean age of 41.09 years (SD = 6.67) and fathers at a mean age of 46.00 years (SD = 7.35). The children of parents with a mental illness had a mean age of 10.39 years (SD = 4.01). Over one half (60%) of parents shared a household with another adult. Nearly one half (49%) of parents lived with the child’s second parent. A total of 12% of the other caregivers had likewise been diagnosed with a mental disorder. Some parents lived alone with their children with no help from another caregiver (14%). The mean net monthly income of a family was 44,110 CZK, approximately 1,790 EUR/1,950 USD, which can be considered slightly lower to medium income for households with children in the Czech Republic. The cumulative family risk index ranged from 0 to 9 in the sample (mean = 3.95, SD = 1.87), indicating variability in the number of risk factors across families. Over one half of the parents (52%) had been previously hospitalized for their mental illness, some more than once a year. Less than one quarter of the parents (23%) had experienced their own parents’ mental illness. More than one half of the parents informed their offspring (58%) and their partners (75%) about their ICD-10 mental disorder diagnosis.

### Relationship between the parent’s diagnosis and the child’s diagnosis

The most prominent primary ICD-10 mental disorder diagnoses in parents were mood (affective) disorders (32%). 34% of parents had comorbid mental disorder diagnoses (Table 1).

**Table 1:** Parents’ mental disorder diagnoses (ICD-10)

**Parents’ ICD-10 mental disorder diagnoses**		N (percentage)
F20–29	Schizophrenia, schizotypal, and delusional disorders	7 (10.77%)
F30–39	Mood (affective) disorders	21 (32.31%)
F40–48	Neurotic, stress-related and somatoform disorders	15 (23.08%)
F50–59	Behavioral syndromes associated with physiological disturbances and physical factors	10 (15.38%)
F90–98	Behavioral and emotional disorders with onset usually occurring in childhood and adolescence	5 (7.69%)
	Other diagnoses	7 (10.77%)
	Parents with any comorbidities	22 (33.85%)

Note: Children’s diagnoses (Table 2) are stratified by gender due to clinically relevant gender differences in child and adolescent psychopathology. Parental diagnoses are presented without stratification because the proportion of fathers in the sample was relatively small (17%). A summary of parental diagnoses stratified by parent gender is provided in the Supplementary Materials 2

Of the 100 participating children, 31 children from 28 families already had an ICD-10 mental disorder diagnosis. The most prevalent ICD-10 mental disorder diagnoses in children were stress-related and somatoform disorders (with the highest prevalence of adjustment disorder diagnosis) (16%), followed by behavioral and emotional disorders with onset usually in childhood or adolescence (with the highest prevalence of ADHD) (12%). Children from eight families (12%) had comorbid ICD-10 mental disorder diagnoses (Table 2).

**Table 2:** Children’s mental disorder diagnoses (ICD-10)

Gender	Age (years)	Total *N* (percentage)	F40–48 diagnosis (*N*)	F90–98 diagnosis (*N*)	Other diagnoses (*N*)
Female	0–8	17 (17.00%)	0	3	0
	9–11	20 (20.00%)	1	2	0
	12–14	14 (14.00%)	8	0	0
	15	11 (11.00%)	2	1	1
Male	0–8	12 (12.00%)	1	2	0
	9–11	13 (13.00%)	1	2	2
	12–14	6 (6.00%)	0	0	0
	15	6 (6.00%)	2	2	0
Not stated	12–14	1 (1.00%)	1	0	0
Total		100 (100.00%)	16	12	3

In a small subset of families (fewer than six), parents and children had diagnoses within the same ICD-10 grouping of stress-related and somatoform disorders. A similar small subset (fewer than six families) had parent–child diagnoses within F90–F98 (behavioral and emotional disorders with onset usually in childhood/adolescence). In the majority of families, parental and child diagnoses fell into different ICD-10 categories (Table 3).

**Table 3:** The relationship between the parent’s and the child’s diagnosis (ICD-10)

Diagnosis (*N*)	Child F40–F48	Child F90–F98	Child other dg.	Families where a child has		Total *N* (percentage)
				No dg.	Any dg.	
Parent F20–F29	2	0	0	5	2	7 (10.77%)
Parent F30–F39	5	5	1	11	10	21 (32.31%)
Parent F40–F48	5	0	0	10	5	15 (23.08%)
Parent F50–F59	0	2	0	8	2	10 (15.38%)
Parent F90–F98	1	5	0	1	4	5 (7.69%)
Parent other dg.	3	0	2	2	5	7 (10.77%)
Total	16	12	3	37	28	65 (100.00%)

Note. Dg. = diagnosis. Counts are presented at the family level. Child diagnostic categories are not mutually exclusive because families may have more than one child with different diagnoses; therefore, a family may appear in more than one child diagnosis column. The column “Any dg.” represents families in which at least one child had a diagnosis and therefore does not equal the sum of the child diagnosis columns

### Parents’ and children’s assessment of the children’s quality of life

Table 4 shows the assessment of children’s quality of life (KIDSCREEN-27) by both children and parents.

**Table 4:** Parents’ and children’s assessment of the children’s quality of life (KIDSCREEN-27)

Subscale	Children raw score (*N* = 40) Mean (SD)	Parent raw score (*N* = 57) Mean (SD)
Physical well-being	12.00 (4.28)	14.32 (4.95)
Psychological well-being	19.68 (6.54)	22.99 (5.68)
Autonomy and parent relations	24.31 (5.61)	27.75 (4.12)
Peers and social support	13.06 (3.65)	13.53 (3.39)
School environment	10.96 (3.84)	12.76 (2.97)

Note. For both versions of KIDSCREEN-27 (parent and child), on the Physical well-being scale, the minimum raw score = 5, the maximum = 25; on the Psychological well-being scale and Autonomy and parent relations scale, the minimum raw score = 7, the maximum = 35; on the Peers and social support scale and School environment scale, the minimum raw score = 4, and the maximum = 20

### Well-being according to the children

In summary, the analyses indicated that children’s self-reported HRQoL (12–18 years) was associated with several family characteristics. For Physical well-being, higher net family income and older parental age were associated with higher scores, whereas having a male parent respondent (i.e., the participating parent with a mental disorder was male) was associated with lower scores. Higher cumulative family risk was associated with lower scores for both Psychological well-being and Autonomy and parent relations. For Peers and social support, children reported higher scores when the parent had a history of hospitalization and when the parent was older. The School environment model did not reach statistical significance.

In the case of children’s Physical well-being score, the overall regression was statistically significant (F = 2.38 (9, 24), *p* = .044). The model accounted for 47% of the variance. The net family income was found to be a significant predictor suggesting that a higher income was related to higher scores in children’s physical well-being. Significant association was also found regarding parent’s age: the older the parent was, the higher the children scored. The opposite relation was found with the parent’s gender: if the parent was male, children scored lower on the physical well-being scale. Parent’s level of education, hospitalization, diagnosis, family risks, number of children and another adult presence were not found to significantly predict children’s physical well-being.

Regarding children’s Psychological well-being score, the overall regression was also statistically significant (F = 4.48 (1, 34), *p* = .041); however, the model explained only 12% of the variability in the score observed. Family risks were found to significantly predict children’s psychological well-being, indicating that the higher the risks, the lower the psychological well-being score. Parent’s gender, age, level of education, hospitalization, diagnosis, net family income, number of children, and another adult presence were not found to significantly predict children’s psychological well-being.

In terms of children’s Autonomy and parent relations, the overall regression analysis also demonstrated a significant statistical finding (F = 3.33 (4, 30), *p* = .002). This model explained 31% of the observed variability. It was observed that family risks significantly influenced children’s Autonomy and parent relations scores, suggesting that higher risks were associated with lower scores. Parental factors such as gender, age, education level, hospitalization, diagnosis, income, number of children, and presence of another adult were not found to be significant predictors of children’s autonomy and parent relations.

In the case of Peers and social support, the overall regression was also statistically significant (F = 5.13 (4, 31), *p* = .003). The model accounted for 40% of the variance. Hospitalization was found to be a significant predictor. If the parent had been hospitalized, the Peers and social support was perceived higher by their children. Also, the older the parent was, the higher the children scored. Parent’s gender, level of education, income, diagnosis, family risks, number of children and another adult presence were not found to significantly predict children’s well-being regarding peers and social support.

For the score at children’s School environment subscale, the overall regression analysis did not yield statistically significant results. Table 5 shows the significant results.

When compared with European normative data for children aged 12–18 years, the scores of children in our sample corresponded approximately to the 10th–25th percentile across KIDSCREEN-27 domains, indicating lower levels of well-being compared with the general population. Percentile values were calculated using European KIDSCREEN-27 normative data available at www.kidscreen.org.

### Children’s well-being according to the parents

Overall, parent proxy HRQoL (8–18 years) reports indicated lower child Physical well-being when the parent was older and had a history of hospitalization, and higher Physical well-being with higher net family income and with more children in the household. For Psychological well-being and Peers and social support, higher cumulative family risk was associated with lower parent-reported scores; male parent respondents reported higher Peers and social support scores. For Autonomy and parent relations, the parental diagnosis category was associated with scores (highest mean in F30–F39).

In the parental questionnaire version, subscale Physical well-being, the regression was statistically significant (F = 6.86 (4, 50), *p* = .000), explaining 35% of the variance in the score. Parent’s age was found as a significant predictor, indicating that older parents tend to perceive their child’s physical well-being more negatively. Additionally, parent’s hospitalization significantly predicted the physical well-being score. If the parent had been hospitalized, the parent assessed the child’s physical well-being lower. The number of children in the family was also found to be a significant predictor indicating that the more children in the family, the better parents perceive children’s physical well-being. Also net family income was found to significantly predict the score: the higher the income was, the higher the score. Parent’s gender, level of education, diagnosis and another adult presence were not found to significantly predict the score.

In the parental questionnaire version, regarding the Psychological well-being subscale score, the linear regression also yielded statistically significant results (F = 9.72 (2, 52), *p* = .000). The model explained 27% of the variance. Notably, family risks emerged as a significant predictor, indicating that higher risk levels were associated with lower scores. However, parent’s gender, age, education level, hospitalization, diagnosis, income, number of children, and presence of another adult were not found to significantly predict parents’ assessments of their children’s psychological well-being.

In the parental version, on the Autonomy and parent relations subscale, the overall regression analysis also revealed significant findings (F = 4.60 (7, 47), *p* = .000), explaining 41% of the variance. Notably, the parent’s diagnosis emerged as a key predictor of their assessment of the child’s Autonomy and parent relations. Parents diagnosed with F30–F39 reported the highest mean score (30.24), indicating they assessed their children most positively in terms of autonomy and parent relations, while the lowest mean score was reported among parents diagnosed with F90–F98 (26.55) and those with other diagnoses (22.9). Parent’s gender, age, level of education, hospitalization, family risks, income, number of children and another adult were not found to significantly predict parent’s assessment of their children’s Autonomy and parent relations score.

Regarding the parental Peers and social support subscale, the overall regression analysis also yielded statistically significant results (F = 4.13 (2, 53), *p* = .022); however, this model explained only 13% of the variance in the score. Parent’s gender emerged as a significant predictor. Parents identifying as male perceived their children to fare better in Peers and social support. Additionally, family risks were found to significantly predict parental scores in this domain, with higher risks associated with lower scores.

In the case of the School environment subscale, the overall regression did not reach statistical significance. Table 5 shows the significant results.

**Table 5:** Multivariate analysis of variables to predict the health-related quality of life according to the children and according to their parents (KIDSCREEN-27)

**Reported by**	**Variable**	***B***	***SE***	***t***	***95% CI***	**F**	**p**	**N**
**Child**	**Physical well-being**							
	Income	0.04	0.02	2.26	[0.01–0.08]		0.033*	34
	Gender Male	-4.73	2.00	-2.37	[-8.65–0.81]		0.027*	34
	Age	0.45	0.15	3.07	[0.16–0.73]		0.005*	34
	**Psychological well-being**							
	Risks	-1.04	0.49	-2.12	[-2.01 – -0.08]		0.042*	36
	**Autonomy and parent relations**							
	Risks	-1.25	0.44	-2.86	[-2.10–0.39]		0.008**	35
	**Peers and social support**							
	Hospitalization	2.93	1.05	2.79	[0.87–4.99]		0.009**	36
	Age	0.20	0.09	2.30	[0.03–0.37]		0.028*	36
**Parent**	**Physical well-being**							
	Age	-0.18	0.09	-2.17	[-0.35 – -0.02]		0.035*	55
	Hospitalization	-3.17	1.15	-2.76	[-5.42 – -0.92]		0.008**	55
	Income	0.04	0.02	2.32	[0.01–0.07]		0.024*	55
	No. of children	1.99	0.77	2.59	[0.48–3.50]		0.013*	55
	**Psychological well-being**							
	Risks	-1.41	0.34	-4.17	[-2.09–0.75]		0.000*	55
	**Autonomy and parent relations**							
	Parent’s diagnosis					5.30 (5,47)	0.001***	55
	**Peers and social support**							
	Gender Male	2.40	1.17	2.05	[0.11–4.69]		0.045*	56
	Risks	-0.46	0.22	-2.09	[-0.89 – -0.03]		0.042*	56

Note.*** = significant at 0.001 level; ** = significant at 0.01 level; * = significant at 0.05 level; F = F statistics in the linear model accompanied by its degrees of freedom; N = number of observations; B = regression coefficient; SE = standard error; t = t-statistic; CI = confidence interval; Income = net family income in CZK; Dg. = Parent’s diagnosis (categorical variable with 6 categories F20–F29, F30–F39, F40–F48, F50–F59, F90–F98, other); Age = Parent’s age; Gender Male = Parent’s gender is male; Risks = the total number of family risk factors (0 no risks, 10 = max risks); Hospitalization = Parent’s hospitalization, 0 = has never been hospitalized, 1 = has been hospitalized at least once

### Differences in children’s and parents’ perceptions

We further examined how parent and child perceptions correspond (Table 6). A positive value means that the child perceives their quality of life more positively than the parent reports (i.e., child self-report > parent proxy). A negative value means that the parent perceives the child’s health-related quality of life more positively than the child does. Negative values were found across all subscales. The biggest discrepancy was in Autonomy and parent relations, where parents tend to assess their children at this scale more positively than how children perceive it.

Across subscales, mean differences (child minus parent) were negative, indicating that parents tended to rate children’s HRQoL more positively than did the children. Discrepancy was largest for Autonomy and parent relations. Analyses of differences included *n* = 33 dyads for Physical well-being, *n* = 34 for Psychological well-being, *n* = 34 for Autonomy and parent relations, *n* = 36 for Peers and social support, and *n* = 35 for School environment. For Physical well-being differences, larger positive discrepancies were associated with parental hospitalization, whereas families with more children showed smaller discrepancies. For Autonomy and parent relations differences, the overall model was significant, but no single predictor reached statistical significance.

**Table 6:** Differences in perception of parents and children in children’s health-related quality of life

**KIDSCREEN- 27 subscale**	Difference Mean (SD)	N
Physical well-being	-0.71 (4.93)	33
Psychological well-being	-1.96 (6.12)	34
Autonomy and parent relations	-3.37 (4.76)	34
Peers and social support	-0.15 (4.82)	36
School environment	-1.30 (4.12)	35

Note. N = number of observations. SD = standard deviation

We also investigated which variables predicted this difference in perception. In the case of physical well-being, the overall regression was statistically significant (F = 4.89 (2, 30), *p* = .015). The model accounted for 25% of the variance. Hospitalization was found to be a significant predictor, suggesting that if the parent had been hospitalized, the score was higher towards positive values, which means that children of parents with the history of hospitalization perceive their physical well-being better, while their parents tend to perceive it more negatively. The number of children was also found to be a significant predictor. The more children in the family, the lower the score was, which implies that the views of children and parents could be aligned, and the parents have a tendency for more positive assessment than their children. In the case of Psychological well-being, Peers and social support and School environment subscales, the regression was not statistically significant. In the case of Autonomy and parent relations, the regression was statistically significant (F = 3.33 (3, 30), *p* = .033). 25% of the variance in the difference can be explained by the independent variables collectively; however, no individual independent variable reached statistical significance. Significant values are displayed in Table 7.

**Table 7:** Multivariate analysis of variables to predict the differences in perception of parents and children in children’s health-related quality of life (KIDSCREEN-27)

**Subscale**	**Variable**	***B***	***SE***	***t***	***95% CI***	**p**	**N**
Physical well-being	Hospitalization	3.49	1.58	2.22	[0.40–6.58]	0.034*	33
	No. of children	-2.05	0.91	-2.25	[-3.83 – -0.27]	0.032*	33

Note.*** = significant at 0.001 level; ** = significant at 0.01 level; * = significant at 0.05 level; N = number of observations; B = regression coefficient; SE = standard error; t = t-statistic; CI = confidence interval; Hospitalization = Parent’s hospitalization, 0 = has never been hospitalized, 1 = has been hospitalized at least once

### Parent’s assessment of threats to their children’s healthy development

Parents’ perceptions of potential threats to their children’s development were assessed using the Parents’ Evaluation of Developmental Status (PEDS). This measure was completed by parents of children aged 21 months to 8 years. Only 18 families completed the scale (approximately 28% of the total sample). Therefore, the results should be interpreted cautiously. Children from half of the families were classified in Path A (high developmental risk; 50.0%), followed by Path B (moderate risk; 38.9%), while children from a small number of families were classified in Path C (low risk; 5.6%) or Path D (no developmental concerns but monitoring recommended; 5.6%).

### Areas in which the children are affected by their parents’ mental illness in relation to lack of financial resources in different areas of the family budget

Financial constraints. More than half of families reported limited resources in at least one budget area, most commonly holidays and developmental/leisure domains (education, hobbies, leisure). A sizable subgroup reported constraints across multiple domains, indicating multi-domain material strain (Table 8).

**Table 8:** Areas where families experience lack of financial resources

**Lack of money for**	N (percentage)
Housing	8 (12.31%)
Food	2 (3.08%)
Clothing	9 (13.85%)
Toiletries	5 (7.69%)
Holidays	37 (56.92%)
Hobbies and leisure for children	15 (23.08%)
Education and leisure for the parent	23 (35.38%)
Repairs	29 (44.62%)
Enough money for everything	20 (30.77%)
No domain-specific financial constraints	21 (32.31%)

Note. Financial constraints were assessed across specific domains: housing, food, clothing, toiletries, holidays, hobbies, education/leisure activities, and household repairs. “No domain-specific financial constraints” indicates families who did not report lack of funds in any of these domains. The item “Enough money for everything” reflects a subjective overall assessment of financial resources

We also summarize the distribution across constrained domains in an additional file (see Additional file 2 Supplementary Table S2 and Supplementary Table S3).

## Discussion

We have examined sociodemographic, risk, and protective characteristics of parents with mental illness and their children in the Czech Republic. Our study offers new insights into these characteristics, although the relatively small sample size of 65 families and 100 children under 18 years of age warrants caution in generalizing the results. Further research with larger and more diverse samples is needed to validate and extend our observations. In terms of the first aim, we found that most of these parents were mothers. Specific to this group was that most participating parents had higher education.

The high participation of mothers and of parents with higher education may reflect greater help-seeking and research participation propensity in these groups, which is consistent with family-focused practice literature [23] but also suggests potential selection effects that limit generalizability [20, 21]. We therefore interpret results cautiously.

In the Czech Republic, as in many European countries, the number of single-parent families is gradually increasing. It was therefore a positive finding that half of the children were growing up with both parents or with a parent and his/her partner. The advantages of two caregivers include their joint contribution to the family budget, whereby if one parent cannot contribute due to mental illness, the family is supported by the other caregiver [34]. Two-caregiver households can buffer financial strain and caregiving demands. Conversely, single parents with mental illness may face heightened role overload and economic hardship [30, 34].

It is clear that for single parents with mental illness, it is much more difficult to manage the household budget. Apart from financial issues, single parents also face other challenges such as emotional issues, managing a wide range of responsibilities on their own, and especially coping on their own with their mental health difficulties. All this can contribute to increased emotional strain and potentially affect their ability to successfully fulfil the parenting role [30]. It is therefore not surprising that children from single-parent families struggled with the issues described above.

In the study, some children were growing up in families where the other caregiver also had an ICD-10 mental disorder diagnosis. These children were exposed to an accumulation of risk factors. Some of the participating parents with mental illness experienced a mental illness in their own parents, which is consistent with the finding that mental health problems often run in families. COPMI often do not have clear information about their parents’ illness: our findings showed that a considerable number of the children had not been informed by their parents about their mental illness. Low awareness among these COPMI can cause complications in their functioning and lead to increased social isolation [21]. Non-disclosure can complicate family communication and children’s coping, and is associated with elevated stigma and social withdrawal in COPMI [15, 21, 28].

The second aim of the study was to examine the relationship between the diagnoses of parents and their children. Our findings align with previously reported results, indicating that children in families with mental illness are at risk for developing a mental disorder, either the same as their parents or a different one. Our pattern—where parent and child diagnoses frequently differ—aligns with prior work documenting both disorder-specific transmission and broader transdiagnostic risk [5, 6].

For instance, we identified five cases where the parents and their children shared a diagnosis, such as ADHD. Then there was a group of children and parents who had disorders from the same ICD-10 diagnostic group of neurotic, stress, and somatoform disorders, whereby the children’s diagnosis was in all cases adjustment disorder. But families with the same or similar diagnosis in parents and children were a minority: in most families, the parents’ and children’s diagnoses differed [5, 6].

This high representation of families where parents and children have different mental disorders indicates that mental disorders in parents lead to a wide range of mental disorders and other negative outcomes in children (multifinality). In our sample, several factors frequently highlighted in the literature as potential risks were present (e.g., parental comorbidity, another caregiver with a mental disorder, and a history of mental illness in the previous generation). While these factors can plausibly contribute to multifinality in offspring outcomes, as suggested by prior research, our study did not test their direct associations with child diagnoses [5].

Given that a proportion of children in our sample were diagnosed with ADHD, it may be useful to consider aspects of the family context, including shared routines, activity patterns, and organizational challenges that may influence daily functioning [41]. Adjustment difficulties in early-to-middle adolescence often follow salient stressors (e.g., family illness, separation, housing/financial stress) and commonly present with interpersonal and school-related impairment [42], all of which are stressors frequently encountered by COPMI.

Globally, children are at the highest risk age for developing any mental disorder around 14.5 years of age, which is consistent with our results, where girls diagnosed with adjustment disorder were mostly around 14 years. In descriptive terms, younger children in our sample appeared to have fewer formal diagnoses; however, we did not conduct statistical tests of age-related differences, and this observation should be interpreted cautiously. Similar to other stress-related and somatoform disorders, adjustment disorder significantly affects social relationships and school performance. COPMI with this diagnosis are therefore likely to experience worsening problems in the family, in peer relationships, and in education [42].

When assessing the children’s quality of life (our third aim), we noted differences in the assessments of parents and children. These comparisons are descriptive only, as no inferential tests were performed for these contrasts. The differences in scores suggest that parents may either have a more optimistic perception of their children’s well-being than the children themselves or that they are unaware of the full range of their children’s difficulties [24].

Some of the participating children were aged 14 years or older, a developmentally sensitive period associated with an increased risk of mental health difficulties. A total of 31% of children in our study already had an ICD-10 mental disorder diagnosis. The children’s age, together with the presence of mental illness in their parents and themselves had an impact on their quality-of-life scores. Not only in comparison to their parents, but also overall, COPMI rated their quality of life as low, which is consistent with other studies reporting that COPMI rated their quality of life as worse than a normative sample of children with parents without a mental illness [43].

Our study highlighted the significant impact of various risk factors on children’s quality of life. One of the most critical findings was the influence of the number of risk factors to which the family is exposed. Families demonstrated a marked impact on the children’s quality of life, underscoring the multifaceted nature of these risk factors. From the children’s perspective, higher family risks were significantly associated with lower psychological well-being. This aligns with previous research indicating that cumulative risk exposure can lead to adverse developmental outcomes. Cumulative exposure to family adversity is known to compromise children’s psychological well-being and family functioning, including autonomy and role negotiations [13, 33].

Children in high-risk families often experience higher degrees of stress, emotional turmoil, and instability, which can hinder their psychological development and overall well-being. Moreover, our results indicated that the presence of multiple risk factors can disrupt family dynamics and the child’s sense of independence. This was evident in the significant impact on children’s Autonomy and parent relations scores, where higher family risks were linked to lower scores. This disruption may be due to the increased caregiving burdens and responsibilities that children in high-risk families often have to assume, which can interfere with their normal development and relationship with parents [13].

The study included an equal number of children with and without siblings. Positive sibling relationships are considered a protective factor for children who are exposed to increased stress and hardship, while dysfunctional sibling relationships can contribute to psychopathology in COPMI [21]. Our results suggest that the children’s quality of life may have been affected not only by the parent’s mental disorder but in many cases also by siblings’ mental disorder, as 31% of the children already had an ICD-10 mental disorder diagnosis [44]. However, it is important to note that the cumulative family risk index used in this study did not distinguish between a child’s own diagnosis and a sibling’s diagnosis; thus, we cannot draw specific conclusions about the unique contribution of sibling psychopathology.

The fact that some parents with personal experience of mental illness are sensitive to their children’s problems was reflected in other assessments. When assessing risks to healthy child development, the parents of children under 8 years rated over one half of the offspring as being at high risk, which indicates a high level of parents’ concern regarding possible developmental and behavioral problems of their offspring.

The fourth aim of our study was to explore the financial situation of FAPMI. In general, people with mental health problems find it harder to manage their financial situation [34]. For FAPMI, this challenge can be even more pronounced due to the need to provide for several people in the household.

Perceived financial constraints were reported across families and may occur at different income levels; these constraints reflect subjective, multi-domain material strain and do not necessarily equate to lower net income. We did not model the association between net income and constraints in this paper; future work should examine whether lower income is associated with more reported constraints [31].

Among these participating families, perceived financial constraints were reflected in limitations across various areas. Some families reported difficulties related to housing and food insecurity, while others faced constraints in areas including holidays, education, repairs, and leisure and hobbies. Due to limited resources for such activities, children’s extracurricular engagement may be restricted, which can negatively affect their academic and social development and may contribute to social isolation [13].

### Practical and clinical implications

In examining the sociodemographic, risk, and protective characteristics of parents with mental illness and their children, our study sheds light on critical aspects that demand attention from mental health professionals and policymakers. Based on the results, we would like to make the following recommendations for practical and clinical implications to provide valuable insights for targeted interventions and contribute to a broader understanding of mental health in families.

1. The high participation of parents with higher education highlights their motivation to seek help for their children. It is crucial to actively engage parents with lower education levels. Mental health professionals can capitalize on the higher motivation of the first group while exploring strategies to involve parents with lower education in preventive interventions for families. Recognizing the motivational factors can guide mental health professionals in formulating intervention strategies that align with the unique characteristics of these families. Empowering parents through targeted communication-focused interventions may enhance overall outcomes. Tailored engagement strategies for parents with lower education may improve reach among families less likely to seek support [23, 30]. Specifically, such strategies may include: using plain-language, visually supported materials; embedding outreach in trusted settings (e.g., primary care, schools, community centers, NGOs); offering flexible appointment times and formats (phone, video, or in-person) and brief pre-appointment engagement calls; providing reminders via SMS or phone calls; partnering with social services to bundle supports (e.g., childcare, transport vouchers); and involving peer supporters or family advocates to normalize help-seeking and reduce stigma.
2. Single-parent families face increased challenges, including financial constraints and emotional strain. Mental health professionals should consider designing support programs that address the specific needs of single parents, focusing on both financial and emotional aspects. Policy recommendations should prioritize support structures for single parents, acknowledging the potential impact of mental health difficulties on their ability to manage parental responsibilities. Targeted interventions can mitigate the emotional strain faced by both parents and children in these families. Single-parent families may benefit from combined financial and psychosocial support [30, 34].
3. Families with a history of mental illness represent vulnerable populations. Recognizing the low awareness among COPMI about their parents’ illness emphasizes the need for improved communication strategies. Mental health professionals should prioritize psychoeducation for parents, ensuring they communicate openly with their children about mental health issues. This can reduce complications in functioning and alleviate the risk of increased social isolation among COPMI. Family-focused psychoeducation and structured communication support can reduce stigma and improve family functioning [14, 15, 23, 28].
4. The high representation of families with diverse mental disorders emphasizes the multifinality of outcomes in children. Mental health professionals should adopt a comprehensive approach, considering factors such as comorbidity and mediating risk factors. Targeted prevention strategies should focus on the diverse range of mental disorders in parents, recognizing the potential impact on children. The incorporation of transdiagnostic approaches can provide efficient and cost-effective interventions. Transdiagnostic preventive approaches map onto multifinality and equifinality in risk transmission [5, 6, 13, 16, 18, 19].
5. Families with financial difficulties face constraints in multiple areas, which may lead to social isolation. Policymakers should consider implementing financial assistance programs and addressing social stigma. Mental health professionals need to collaborate with policymakers to ensure that families receive adequate financial support. Targeted interventions can address the specific challenges faced by children in terms of education, leisure, and social interactions. Addressing socioeconomic hardship is a key component of mental health equity [31, 32, 33].

By aligning our findings with these practical and clinical implications, we contribute to the ongoing dialogue on supporting families affected by parental mental illness. Situating our study within the larger framework of existing research enhances its significance, offering a comprehensive perspective for mental health professionals and policymakers alike.

### Recommendation for preventive action based on our results

It is essential to engage FAPMI and COPMI in early preventive interventions that offer support and can, if need be, refer them to other specialist services. COPMI support is essential given the cumulative impact of parental mental illness on their well-being. Early identification of COPMI, particularly in younger age groups, can help prevent future social and emotional problems and the need for more complex mental health care.

A family-focused approach also helps develop communication within the family and supports parents in improving their parenting competencies. A comprehensive approach including mental health services, financial assistance, and educational support is necessary to address the family dynamics and meet the multifaceted needs of FAPMI. Involvement of parents in family-centered interventions promotes sibling relationships and supports the overall well-being of children in these families.

To address the financial challenges of FAPMI and COPMI, targeted support from state agencies is needed to promote a more inclusive and responsive society. Mental health literacy is essential and better awareness of problems faced by FAPMI and COPMI enables professionals to provide meaningful support.

### Further research

Future research should further examine the different experiences of families across socioeconomic backgrounds in relation to protective mechanisms. Understanding how financial constraints affect children’s access to education, leisure, and social activities is essential to inform the development of interventions that target the specific needs of these vulnerable populations. Future research should also address factors that influence parents’ awareness of and sensitivity to children’s problems, particularly in terms of mental health. Research should also investigate families’ experiences of stigma. Examination of the influence of cultural factors on family dynamics would further contribute to a comprehensive understanding of this issue.

### Strengths and limitations

In interpreting these findings, several limitations should be considered. The sample size was modest and recruited via clinician referral across diverse settings, which may limit generalizability and introduce selection bias, such as the overrepresentation of mothers and parents with higher education. The data collection period, which commenced during the COVID-19 pandemic, may have further affected recruitment and institutional participation. Additionally, the study was cross-sectional; therefore, causal inferences cannot be drawn. Certain measures were restricted by age for ethical reasons (e.g., KIDSCREEN self-report for children aged ≥ 12 years and parent proxy for those aged ≥ 8 years), and findings related to PEDS were primarily descriptive due to the small subsample size.

Furthermore, our cumulative family risk index was a formative composite; while conceptually related covariates like net income were included for interpretability, residual construct overlap remains possible. We did not systematically track reasons for exclusion or the number of families contributed by each specific institution, which may have influenced the representativeness of the sample. Regarding the instruments used, the sociodemographic questionnaire was completed by parents only; thus, we did not obtain children’s perspectives on risk and protective characteristics. We also did not collect data on parental employment status, duration of diagnoses, or perceived stigma and discrimination, which could have provided additional contextual depth. Finally, although we report perceived financial constraints, we did not model their association with net income; these constraints should be interpreted as subjective, multidomain material strain that can occur across various income levels.

## Conclusion

This study has confirmed the previously reported existence of multifactorial associations between parental mental illness and mental disorders in children. Its results support the need for a targeted transdiagnostic prevention for FAPMI and COPMI within a family-centered approach.

We believe this study illustrates the need for a comprehensive approach that includes focus on improvement of health promotion, targeted prevention, and resources to address the diverse needs of families affected by parental mental illness. The findings offer valuable insights for mental health professionals and policymakers to design interventions that would promote the well-being of parents and children and ultimately contribute to breaking the cycle of intergenerational mental health problems in families.

By situating these findings within the Czech context and across diverse service settings, this study underscores the value of family-centered, transdiagnostic preventive strategies tailored to local systems and resource profiles [13, 16, 18, 19, 23].

## Supplementary Information

Below is the link to the electronic supplementary material.

Supplementary Material 1: This file contains the English version of the Sociodemographic and risk & protective characteristics questionnaire developed specifically for this research

Supplementary Material 2: This file contains supplementary tables

## Data Availability

The original contributions presented in this study are included in the article and supplementary material. Requests for further information or specific data can be directed to the corresponding author.

## Abbreviations

FAPMI: Families with a parent with a mental illness
COPMI: Children of parents with mental illness
ICD-10: International Classification of Diseases, 10th edition
ADHD: Attention deficit hyperactivity disorder
