# Supplementary material for: Characteristics of parents with a mental illness and their minor children in a study sample from the Czech Republic: a cross-sectional study
Source: BMC Psychiatry. 2026 May 16;26:442. doi: 10.1186/s12888-026-08118-6 (PMC13231712; doi:10.1186/s12888-026-08118-6)
Supplement: Supplementary file 1 — Supplementary Material 1: This file contains the English version of the Sociodemographic and risk & protective characteristics questionnaire developed specifically for this research [file 12888_2026_8118_MOESM1_ESM.pdf]

Sociodemographic questionnaire

Fill in the code that your therapist gave you:

What is your gender?

- ☐ female
- ☐ male
- ☐ other/don't want to mention

How old are you?

Enter the number.

What is your highest level of education?

- ☐ Elementary - low
- ☐ Vocational - medium
- ☐ Secondary - medium
- ☐ Higher - high
- ☐ University - high

What is your psychiatric diagnosis?

Please provide the diagnosis name as determined by a doctor.

Do you have any other (comorbid) psychiatric diagnoses?

Please provide the diagnosis name as determined by a doctor.

How often are you hospitalized for mental illness?

- ☐ I have never been hospitalized
- ☐ Only exceptionally
- ☐ Once or twice a year
- ☐ More often

How many adults live in your household?

Enter the number.

Now we will ask you for basic information about all the children in your care.

How many children do you have in your care?

- ☐ 1
- ☐ 2
- ☐ 3
- ☐ 4
- ☐ 5 and more

1. child - gender:
- ☐ female
- ☐ male
- ☐ other/don't want to mention

1. child - age:

Enter the number.

1. child – psychiatric diagnosis, if any:

If your child does not have a psychiatric diagnosis, write no.

2. child - gender:
- ☐ female
- ☐ male
- ☐ other/don't want to mention

2. child - age:

Enter the number.

2. child – psychiatric diagnosis, if any:

If your child does not have a psychiatric diagnosis, write no

3. child - gender:
- ☐ female
- ☐ male
- ☐ other/don't want to mention

3. child - age:

Enter the number.

3. child – psychiatric diagnosis, if any:

If your child does not have a psychiatric diagnosis, write no

4. child - gender:
- ☐ female
- ☐ male
- ☐ other/don't want to mention

4. child - age:

Enter the number.

4. child – psychiatric diagnosis, if any:

If your child does not have a psychiatric diagnosis, write no

5. child - gender:
- ☐ female
- ☐ male
- ☐ other/don't want to mention

5. child - age:

Enter the number.

5. child – psychiatric diagnosis, if any:
If your child does not have a psychiatric diagnosis, write no

More children:

Who, apart from you, is taking care of your child/children:

- ☐ The second parent of the child/children with whom I live in the same household
- ☐ The second parent of the child/children with whom I do not live in the same household
- ☐ The partner who is not the parent of the child/children
- ☐ One or both grandparents
- ☐ Broader family (e.g. aunt, uncle, etc.)
- ☐ Someone else
- ☐ Noone else

Does the other caregiver have a psychiatric diagnosis?

- ☐ Yes
- ☐ No
- ☐ I do not know

Does your child/do your children know about your mental illness?

- ☐ Yes
- ☐ No
- ☐ I am not sure

Does the other caregiver know about your mental illness?

- ☐ Yes
- ☐ No
- ☐ I am not sure

Does one of your parents have a psychiatric diagnosis?

- ☐ Yes, both parents
- ☐ Yes, only mother
- ☐ Yes, only father
- ☐ No
- ☐ I do not know

Enter the name of your mother's psychiatric diagnosis:
If you do not know the diagnosis, write "don't know". If the mother does not have a diagnosis, write "none". You can provide other important related information.

Enter the name of your father's psychiatric diagnosis:

If you do not know the diagnosis, write "don't know". If the mother does not have a diagnosis, write "none". You can provide other important related information.

What is the total net monthly income of your household? Enter in CZK (number)

The net income of the household includes the income of household members from employment, business, income from renting real estate, paid state social assistance benefits and benefits in material need, income from capital assets.

For which types of expenses do you usually not have enough money?

- ☐ Housing (Rent/mortgage, payments for housing-related services, e.g. electricity, gas, etc.)
- ☐ Food
- ☐ Clothing
- ☐ Toiletries
- ☐ Holidays
- ☐ Hobbies and leisure for children
- ☐ Education and leisure for you
- ☐ Repairs
- ☐ Other
- ☐ I have enough money for everything

If you wish, leave us your comments or explanations of some answers. You can provide other important related information.
