# Supplementary material for: Characteristics of parents with a mental illness and their minor children in a study sample from the Czech Republic: a cross-sectional study
Source: BMC Psychiatry. 2026 May 16;26:442. doi: 10.1186/s12888-026-08118-6 (PMC13231712; doi:10.1186/s12888-026-08118-6)
Supplement: Supplementary file 2 — Supplementary Material 2: This file contains supplementary tables [file 12888_2026_8118_MOESM2_ESM.pdf]

Additional File 2

Supplementary Table S1. Family cumulative risk index: domains and scoring

Domain	Indicator	Scoring
Parental clinical severity	Psychiatric comorbidity	1
	Frequent psychiatric hospitalization (≥1–2 times per year)	1
Family psychiatric burden	At least one child with an ICD-10 mental disorder	1
	At least one grandparent (parent of the respondent) with a mental disorder	1
	Second caregiver with a mental disorder	1
Caregiving support	Co-residing second parent sharing caregiving responsibilities	0
	Caregiving support available but without a co-residing second parent (e.g., non-resident partner, grandparents, extended family)	1
	No additional caregiver reported	2
Diagnosis awareness	Second caregiver unaware of the respondent's mental disorder	1
	Children unaware of the respondent's mental disorder	1
Financial strain across basic and developmental needs	Lack of funds reported in 1–2 domains*	1
	Lack of funds reported in ≥3 domains*	2

Note. Financial domains included housing, food, clothing, toiletries, holidays, hobbies, education/leisure activities, and household repairs. The family cumulative risk index is a formative measure in which individual indicators define cumulative adversity rather than reflect a single latent construct. Scores range from 0 to 10, with higher scores indicating greater cumulative family risk.

Supplementary Table S2. Distribution of families by number of constrained budget domains

Number of constrained domains	N	%
0 domains (no constraints)	21	32.31
1 domain	10	15.38
2 domains	15	23.08
3 domains	6	9.23
4 domains	3	4.62
5 domains	4	6.15
More than 6 domains	6	9.23

Supplementary Table S3. Financial constraints across budget domains

Financial constraints category	N	%
No constrained domains (0)	21	32.31
Lack of funds reported in 1–2 domains*	25	38.46
Lack of funds reported in ≥3 domains*	19	29.23
Total	65	100.00

*Budget domains included housing, food, clothing, hygiene, holidays, hobbies, education/leisure activities, and household repairs.

Note. Constrained budget domains were derived from domain-specific items (housing, food, clothing, hygiene, holidays, hobbies, education/leisure activities, and household repairs). This supplementary table summarizes the number of families with financial constraints across multiple domains (0, 1–2, ≥3). The global item *"enough money for everything"* reported in Table 8 reflects a subjective overall assessment and is therefore not included in this domain-based summary.

Supplementary Table S4. Parents' mental disorder diagnoses (ICD-10) stratified by gender

Parents'	ICD-10 mental disorder diagnoses	Female N	Male N
F20–29	Schizophrenia, schizotypal, and delusional disorders	6	1
F30–39	Mood (affective) disorders	18	3
F40–48	Neurotic, stress-related and somatoform disorders	11	4
F50–59	Behavioral syndromes associated with physiological disturbances and physical factors	10	–
F90–98	Behavioral and emotional disorders with onset usually occurring in childhood and adolescence	4	1
	Other diagnoses	5	2
	Parents with any comorbidities	19	3
